# Influence of Nutritional Interventions on Functional Outcomes in Stroke Rehabilitation: A Systematic Review

**DOI:** 10.7759/cureus.53711

**Published:** 2024-02-06

**Authors:** Nikhil Deep Kolanu, Sheraz Ahmed, Munara K Kerimkulova, Mikołaj Stańczak, Guillermo de Jesus Aguirre Vera, Naimatullah Shaikh, Anirudh Reddy Addula, Meher Cheran, Srikar P Chilla, Sergio Rodrigo Oliveira Souza Lima, Abdullah Shehryar, Abdur Rehman

**Affiliations:** 1 General Surgery, China Medical University, Shenyang, CHN; 2 Medicine, Islamic International Medical College, Islamabad, PAK; 3 Internal Medicine, Kyrgyz State Medical Academy, Bishkek, KGZ; 4 Medicine, Kharkiv Institute of Medicine and Biomedical Sciences, Kharkiv, UKR; 5 Medicine, Ignacio A. Santos School of Medicine, Monterrey, MEX; 6 Internal Medicine, Chandka Medical College, Larkana, PAK; 7 Internal Medicine, American International Medical University, Chicago, USA; 8 Medicine, CARE Hospitals, Hyderabad, IND; 9 School of Health Sciences, University of East London, London, GBR; 10 Plastic Surgery, Hospital da Bahia, Salvador, BRA; 11 Dermatology, Allama Iqbal Medical College, Lahore, PAK; 12 Surgery, Mayo Hospital, Lahore, PAK

**Keywords:** holistic care, muscle mass maintenance, protein intake, energy intake, nutritional risk, sarcopenia, functional outcomes, nutritional interventions, stroke rehabilitation

## Abstract

Stroke, a major cause of disability worldwide, necessitates comprehensive rehabilitation, with nutrition playing a pivotal role in recovery. Our systematic review assesses the impact of nutritional interventions on stroke survivors' rehabilitation. Adhering to Preferred Reporting Items for Systematic Reviews and Meta-Analyses (PRISMA) guidelines, we systematically searched PubMed, Embase, Web of Science, and Scopus, using keywords related to stroke rehabilitation and nutrition. Studies were selected based on criteria emphasizing dietary interventions and their effect on functional recovery in stroke patients. The review involved detailed data extraction and synthesis, covering study design, participant characteristics, interventions, and outcomes.

Five studies met our inclusion criteria, encompassing longitudinal and prospective studies, retrospective cohorts, and randomized controlled trials. These studies highlighted the importance of early nutritional assessment, particularly for sarcopenic patients, and the role of energy and protein intake soon after a stroke. Findings indicated high nutritional risk correlated with poorer functional outcomes and increased inflammation. Tailored dietary support appeared beneficial for muscle mass maintenance and overall functional recovery, especially in older patients. Our review emphasizes the critical role of nutritional interventions in stroke rehabilitation. It suggests that personalized nutritional strategies can positively impact functional recovery, notably in older and nutritionally vulnerable stroke survivors. These insights underscore the necessity of integrating dietary assessments and interventions into standard stroke rehabilitation protocols, advocating a holistic approach to patient care.

## Introduction and background

Stroke, a medical condition caused by the interruption of blood supply to the brain, is a major global health challenge. It is the leading cause of long-term disability worldwide, profoundly affecting individuals, families, and healthcare systems [1]. Globally, stroke represents a significant public health burden, being the primary contributor to neurological disability-adjusted life years (DALYs). In 2016, it was responsible for 42.2% of these DALYs, underscoring its impact on global health [2]. Despite significant advancements in acute stroke treatment, the journey to recovery often requires comprehensive rehabilitation to restore function [3]. Among the various therapeutic approaches, nutritional interventions are increasingly recognized as essential. These interventions not only support general health but may also enhance recovery outcomes in stroke survivors [4], with emerging research focusing on the role of nutrition in neuroplasticity and the mechanisms of recovery post stroke [5].

A holistic approach that includes nutritional assessments and interventions is pivotal in stroke rehabilitation [6]. Current studies emphasize the importance of specific nutrients, dietary patterns, and supplementation in stroke recovery, indicating the necessity of tailored nutritional strategies. These strategies are aimed at supporting neural repair and enhancing the recovery process, thus playing a critical role in improving the outcomes for stroke survivors [7-9].

This review adopts a structured methodology to explore the impact of nutritional interventions on functional outcomes in stroke rehabilitation. Through a critical analysis of diverse research, including cohort studies and randomized trials, this review aims to identify dietary factors that influence the recovery of motor functions, cognitive abilities, and daily living activities in stroke survivors [10].

Furthermore, this review offers evidence-based clinical recommendations, guiding healthcare professionals in optimizing rehabilitation protocols to improve the quality of life of stroke survivors [11]. The focus is on examining and synthesizing evidence regarding the role of nutrition in functional recovery post stroke. By integrating findings from various studies, we aim to understand the role of nutrition in enhancing motor skills, cognitive functions, and the performance of daily activities post stroke. This comprehensive review seeks to identify key nutrition-related factors in rehabilitation, inform clinical practices, and develop patient-centric strategies that contribute to the long-term well-being and independence of stroke survivors.

## Review

Materials and methods

Search Strategy

Our systematic review investigated the relationship between nutrition and rehabilitation outcomes in stroke patients. Following Preferred Reporting Items for Systematic Reviews and Meta-Analyses (PRISMA) guidelines, we developed a thorough search strategy for relevant literature in critical databases like PubMed, Embase, Web of Science, and Scopus.

We used specific keywords such as "Nutritional Status," "Stroke Rehabilitation," "Sarcopenia," and others, employing Boolean operators for diverse research perspectives. For instance, "Nutritional Status AND Stroke Rehabilitation AND Functional Recovery" helped narrow pertinent studies. Our search spanned from the databases' inception to November 2023. This approach led us to a broad range of studies

We critically analyzed. We aimed to consolidate the current understanding of nutrition's impact on stroke rehabilitation to enhance patient care and therapy approaches.

Eligibility Criteria

In our systematic review exploring the correlation between nutrition and rehabilitation outcomes in stroke patients, we rigorously established specific eligibility criteria to guarantee the inclusion of pertinent and high-quality studies. These criteria were meticulously crafted to align with the research's objectives and to adhere to the principles of a comprehensive and impartial literature review, in accordance with the PRISMA guidelines.

Our inclusion criteria encompassed a wide array of study designs, including longitudinal, prospective, and retrospective cohort studies and randomized controlled trials. This expansive approach aimed to offer a holistic perspective on the subject matter from various research angles. Moreover, we focused on studies involving adult stroke patients during their post-stroke rehabilitation phase to ensure the relevance of the data to our research question.

In terms of interventions and comparisons, our inclusion criteria comprised studies that investigated nutritional interventions or assessments within the context of stroke rehabilitation. This encompassed research on dietary patterns, nutritional supplementation, and evaluations of nutritional status and their impacts on rehabilitation outcomes. Additionally, we prioritized studies that measured functional outcomes related to stroke rehabilitation, such as the recovery of motor functions, activities of daily living (ADL), cognitive abilities, and overall patient well-being.

To maintain the currency of our review, we considered studies published from the inception of the databases until November 2023, facilitating a thorough and up-to-date examination of the literature. Furthermore, to ensure the feasibility of a comprehensive analysis and synthesis of the findings, we restricted our inclusion criteria to studies published in English.

Our exclusion criteria were designed to exclude studies with non-relevant populations, namely, those not involving stroke patients undergoing rehabilitation or those not specifically addressing post-stroke rehabilitation. We also excluded research that did not explore the connection between nutrition and stroke rehabilitation outcomes, as well as study designs such as editorials, commentaries, case reports, and reviews, focusing solely on original research studies. To maintain the quality and reliability of our analysis, we excluded studies with incomplete data or unclear methodologies. Lastly, due to language constraints, we excluded studies not published in English.

By rigorously adhering to these eligibility criteria, we ensured that our systematic review was focused, relevant, and comprehensive. This approach allowed us to provide a clear and accurate assessment of the current state of knowledge in the field of nutrition and stroke rehabilitation, contributing valuable insights to the scientific community and healthcare practitioners alike.

Data Extraction

In our systematic review of nutrition and rehabilitation in stroke patients, we employed a detailed data extraction method to ensure accurate, comprehensive analysis. Initially, we screened articles by titles and abstracts. Two independent reviewers assessed each for relevance, classifying them as "relevant," "not relevant," or "potentially relevant" based on study focus, population, and alignment with our objectives.

Next, we examined full texts of potentially relevant articles. Using a standardized Microsoft Excel form, two reviewers conducted this thorough review. The form ensured uniform data collection, covering author names, publication year, study country, participant demographics, study setting, design, methodologies, outcome measures, and primary findings. Reviewers applied pre-set inclusion and exclusion criteria independently. Discrepancies were resolved by consulting a third reviewer, ensuring a consensus-driven approach. This meticulous process captured extensive information, enabling an in-depth analysis of how nutrition affects stroke rehabilitation. Our systematic approach ensured consideration of all relevant data, enhancing the thoroughness and validity of our review.

Data Analyses and Synthesis

In our systematic review of the impact of nutritional status on stroke rehabilitation, we employed a narrative synthesis method for data analysis. This approach handled the diversity in study designs and outcomes, enabling a cohesive interpretation of how nutrition affects stroke recovery. We closely examined the relationship between nutritional assessment tools (Geriatric Nutritional Risk Index (GNRI), Prognostic Nutritional Index (PNI), Controlling Nutritional Status (CONUT)) and rehabilitation outcomes across various stages of stroke recovery, such as functional recovery, ADL performance, and cognitive status.

We also considered factors such as age, stroke type, and comorbidities, assessing their interaction with nutritional status. Our findings, contextualized within current literature and practices, emphasized the importance of early dietary assessment and intervention. We identified research gaps, suggesting future study directions in less-explored areas. This synthesis provided valuable insights into the significance of nutrition in stroke rehabilitation, informing clinical practice and enhancing understanding in the field.

Results 

Study Selection Process

In conducting our systematic review on nutrition and stroke rehabilitation outcomes, we implemented a detailed study selection process aligned with PRISMA guidelines, involving several stages to refine our pool of studies. Initially, our comprehensive database search yielded 289 records. We removed nine duplicates, reducing the count to 280 potential studies. In the screening phase, we excluded 189 studies that did not meet our research criteria, leaving 91 for more detailed evaluation.

**Figure 1:**
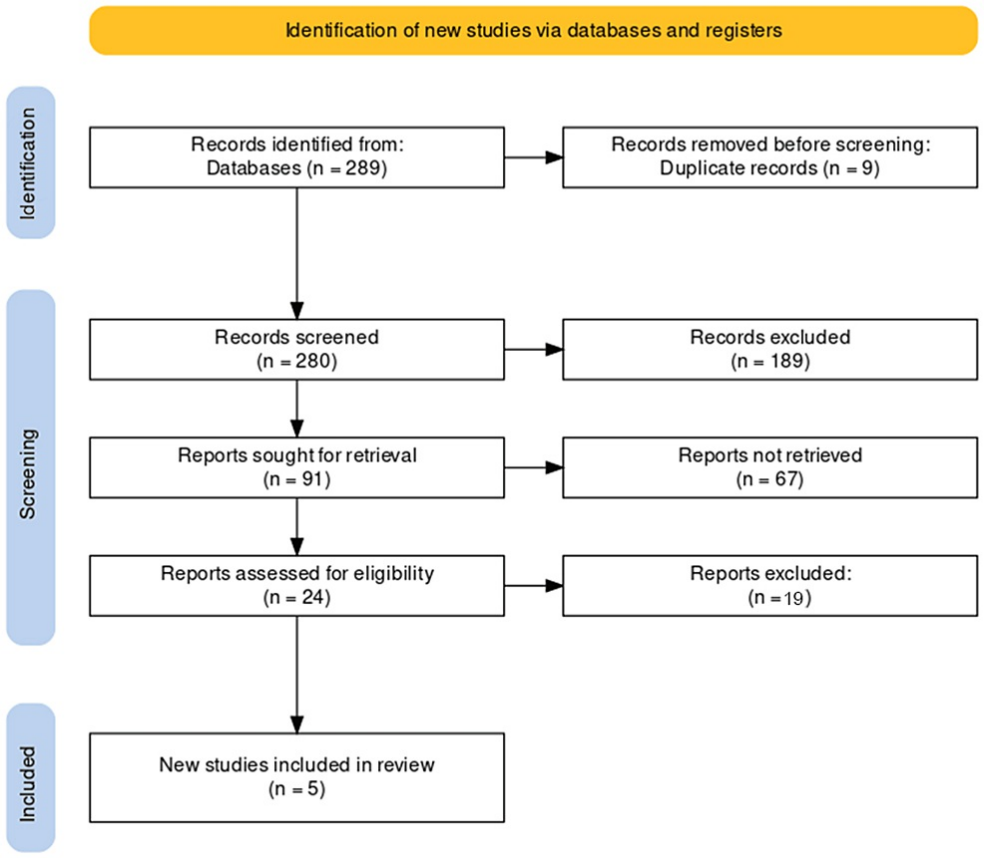
PRISMA flow diagram of the selection of studies for inclusion in the systematic review. PRISMA: Preferred Reporting Items for Systematic Reviews and Meta-Analyses

Out of these, 67 were inaccessible and thus excluded from further assessment. We thoroughly examined the remaining 24 studies against our pre-defined eligibility criteria. This rigorous process led to the exclusion of 19 studies due to issues such as insufficient data, inappropriate study design, or irrelevant outcomes, resulting in a final selection of five studies deemed appropriate for our review. This meticulous selection process, represented in our flowchart (Figure 1), ensured a precise and methodical approach to study inclusion, adhering to established systematic review standards.

Characteristics of Selected Studies

The systematic review incorporated five studies that collectively aimed to understand the interplay between nutritional status and functional outcomes in stroke rehabilitation scenarios. The studies varied in design, combining longitudinal, prospective, and retrospective cohort studies and randomized controlled trials. The participant profiles ranged from post-stroke patients in rehabilitation settings to those in acute care, with sample sizes varying from 61 to 245 individuals. A summary of all the studies is provided in Table 1.

**Table 1:** Summary of studies included in the systematic review. BIA: bioelectrical impedance analysis; MNA®-SF: Mini Nutritional Assessment-Short Form; BMI: body mass index; GNRI: Geriatric Nutritional Risk Index; ADL: activities of daily living; FIM: Functional Independence Measure; MUAC: mid-upper arm circumference; PNI: Prognostic Nutritional Index; CONUT: Controlling Nutritional Status; BI: Barthel Index; mRS: Modified Rankin Scale; TCT: Timed Chair Test; SBS: Stroke-Specific Barthel Index; SPMSQ: Short Portable Mental Status Questionnaire; CRP: C-reactive protein

Authors	Objective	Study design	Participants	Methods	Key findings	Conclusion
Siotto et al. [11]	To assess the relationship between nutritional status, food consumption, and sarcopenia in post-stroke rehabilitation	Longitudinal, prospective study	61 post-stroke patients were admitted for rehabilitation	Assessment of sarcopenia using handgrip test and BIA; evaluation of nutritional status using MNA®-SF, BMI, and GNRI; analysis of food consumption and plate waste	Sarcopenic patients exhibited worse muscle quality, lower nutritional status scores, and higher food waste compared to non-sarcopenic patients. Sarcopenia was associated with poorer functional recovery	Accurate diagnosis of sarcopenia and evaluation of nutritional status are critical for designing targeted interventions in post-stroke patients to improve outcomes
Sato et al. [12]	To evaluate the impact of energy and protein intake during the first week of hospitalization on home discharge and ADL in acute stroke patients	Retrospective cohort study	201 stroke patients were admitted to an acute care hospital in Japan	Evaluate energy and protein intake during the first week of hospitalization; analyze variables related to discharge destination and ADL at discharge	Higher energy and protein intake in the first week was associated with an increased likelihood of home discharge and higher ADL scores at discharge. Energy intake >20.7 kcal/kg/day indicated better outcomes	Early nutritional intake, specifically energy intake, is an essential predictor of home discharge and functional recovery in acute stroke patients
Irisawa and Mizushima [13]	To clarify the effects of body composition and nutritional status on ADL recovery during stroke rehabilitation	Prospective study	179 patients admitted to stroke rehabilitation units in Japan	Use of BIA and GNRI to assess muscle mass and nutritional status; evaluation of ADL using the FIM	High muscle rate, phase angle, and GNRI were significantly associated with improved motor FIM items at four weeks, indicating that muscle mass maintenance and nutritional management are essential for functional recovery in stroke patients	Nutritional status and muscle mass at the start of intensive stroke rehabilitation significantly influence functional recovery, underscoring the importance of nutritional management and rehabilitation for maintaining muscle quality
Ha et al. [14]	To assess if individualized nutritional support alters body composition in older acute stroke patients and examine the correlation between methods for assessing body, fat, and fat-free mass	Randomized controlled trial	Acute stroke patients >65 years at nutritional risk, randomized to individualized nutritional treatment (n=58) or routine nutritional care (n=66)	Patients received either individualized, energy- and protein-rich supplementation or routine care in the hospital. Body composition was assessed with anthropometry and bioelectrical impedance over three months	During the first week in the hospital, weight loss was smaller in the intervention group. After three months, weight and fat loss were significant in both groups, with women in the intervention group showing smaller losses than controls. MUAC correlated highly with BMI	Individualized nutritional support was beneficial for maintaining body mass and composition in the first week, especially among women. MUAC can be a helpful measure when BMI cannot be obtained. Regular assessment of dietary intake and nutritional status is recommended post discharge for older stroke patients at nutritional risk
Di Vincenzo et al. [15]	To determine the relationship between nutritional risk and functional status in stroke patients at admission to a rehabilitation unit	Single-center retrospective cross-sectional study	245 post-acute stroke patients (47% women; 81.6% ischemic stroke), aged 69.7±12.8 years	Nutritional risk was assessed using GNRI, PNI, and CONUT scores. Functional status was evaluated using BI, mRS, TCT, SBS, and cognitive status with SPMSQ, laboratory tests for CRP, fibrinogen, and D-dimer	A high prevalence of nutritional risk was detected, greater with GNRI, especially in patients aged ≥75 years. High nutritional risk was associated with poorer performance on all functional tests and higher CRP, fibrinogen, and D-dimer levels	High nutritional risk, as evaluated with GNRI, PNI, and CONUT scores, was common in stroke patients at admission to a rehabilitation unit and was associated with poor functional status and higher inflammation. Nutritional risk should be regularly assessed in these patients

In terms of methodologies, the studies employed a diverse array of assessment tools to evaluate the nutritional and functional status of stroke patients. These included handgrip tests, which are a straightforward and effective way to measure muscle strength; bioelectrical impedance analysis (BIA), a technique that estimates body composition such as muscle mass and fat percentage; and various nutritional assessment tools. Among these tools were the Mini Nutritional Assessment-Short Form (MNA-SF), which is a quick, validated screening tool used to identify nutritional risk, particularly in the elderly; the body mass index (BMI), a widely used measure to classify underweight, normal weight, overweight, and obesity based on height and weight; and the GNRI, which is a simple and validated tool to assess nutritional risk in older adults.

The evaluation of food consumption was also integral to these studies, providing insights into the dietary patterns and specific nutritional intake of stroke patients. This information is crucial for tailoring nutritional interventions to meet individual needs.

Functional outcomes were measured using established scales such as the Functional Independence Measure (FIM) and the Barthel Index (BI). The FIM is a comprehensive tool that assesses a patient's level of disability and indicates how much assistance is required for the individual to carry out ADL. The BI, on the other hand, measures the performance in 10 basic ADL, indicating the patient's independence.

Additionally, the studies incorporated assessments of cognitive status, which is vital in stroke rehabilitation, as cognitive impairments can significantly impact recovery and the effectiveness of rehabilitation strategies. Importantly, the studies included laboratory tests for inflammatory markers and other predictive biomarkers. Inflammatory markers, such as C-reactive protein (CRP), fibrinogen, and D-dimer, are crucial in understanding the inflammatory response post stroke, which can significantly influence recovery and rehabilitation outcomes. Elevated levels of these markers can indicate ongoing inflammation, which is known to adversely affect stroke recovery. Other predictive biomarkers included in the studies were selected to provide insights into the overall health and functional status of stroke patients. These biomarkers can include blood lipids, glucose levels, and other metabolic indicators, which help in assessing the risk of comorbidities and complications that could affect the rehabilitation process.

By utilizing these varied methodologies, the studies provided a comprehensive analysis of the complex interplay between nutrition, functional and cognitive recovery, and overall health in the context of stroke rehabilitation. The selected studies were conducted across different countries, providing a diverse international perspective on the nutritional challenges faced by stroke patients undergoing rehabilitation. The research highlights the significance of early dietary assessment and intervention in improving rehabilitation outcomes and the potential benefits of individualized dietary support in maintaining body composition post stroke, particularly in older patients at nutritional risk. These characteristics form the basis for further discussion and analysis within the systematic review.

In assessing the quality of the studies included in this systematic review, a detailed evaluation was conducted using the Newcastle-Ottawa Scale (NOS). This scale is specifically designed for evaluating the quality of non-randomized studies, particularly in the context of systematic reviews and meta-analyses. The NOS criteria focus on three primary dimensions: the selection of study groups, the comparability of these groups, and the ascertainment of either the exposure or the outcome of interest for case-control or cohort studies, respectively. Each study was meticulously assessed on these parameters, with scores assigned in each category. The total score, with a maximum of 9, provides an overall indication of the study's quality. This rigorous assessment ensures that the systematic review incorporates evidence from sources that meet high standards of research methodology and data reliability. Table 2 provides the details of the quality assessment of all the included studies.

**Table 2:** Quality assessment of included studies using the NOS. This table summarizes the evaluation of each study based on selection criteria, comparability, and outcome assessment, providing an overall quality score out of 9. NOS: Newcastle-Ottawa Scale

Study reference	Study design	Selection (4)	Comparability (2)	Outcome (3)	Total (9)	Key quality notes
Siotto et al. [11]	Longitudinal, prospective study	★★★★	★★	★★★	9/9	Well-defined cohort, excellent follow-up, clear outcomes
Sato et al. [12]	Retrospective cohort study	★★★★	★★	★★☆	8/9	Well-defined cohort, clear ascertainment of exposure
Irisawa and Mizushima [13]	Prospective study	★★★★	★★	★★★	9/9	Explicit cohort definition, good comparability
Ha et al. [14]	Randomized controlled trial	★★★☆	★★	★★☆	8/9	Good cohort definition, some uncertainty in the representation
Di Vincenzo et al. [15]	Single-center retrospective cross-sectional study	★★★★	★★	★★★	9/9	Well-defined cohorts, clear outcome assessment

Discussion

This review emphasizes the pivotal role of nutrition in shaping functional outcomes during stroke rehabilitation, a finding echoed across various studies. The research by Siotto et al. [11], Sato et al. [12], Irisawa and Mizushima [13], Ha et al. [14], and Di Vincenzo et al. [15] collectively illuminates the multifaceted impact of nutrition on post-stroke recovery pathways. Siotto et al. [11] underscore the necessity of precise nutritional evaluations, stressing the potential benefits of omega-3 fatty acids on neuroplasticity, a notion supported by the findings of Spencer et al. [4] and Hankey [6]. The significance of these fatty acids extends beyond basic nutritional support, influencing the complex mechanisms of brain repair and functional restoration post stroke.

Sato et al. [12] demonstrate the predictive value of early energy and protein intake in stroke recovery, aligning with the research of Daniel et al. [7] and Nooyens et al. [16,17]. This underscores the importance of a nutrient-rich diet, not just for sustaining basic health needs but also for enhancing cognitive resilience and recovery efficiency. Nutritional interventions in the early stages post stroke play a critical role in setting the trajectory for long-term recovery, particularly in terms of cognitive and functional abilities.

The emphasis on muscle mass maintenance through nutrition for functional recovery by Irisawa and Mizushima [13] highlights the direct link between physical strength, mobility, and nutritional status. This connection is vital, as muscle mass and strength are crucial determinants of functional independence and quality of life post stroke. Ha et al. [14] further illustrate the criticality of individualized nutritional support, particularly for older stroke patients, advocating for the use of practical tools like the mid-upper arm circumference (MUAC) for effective assessment.

Di Vincenzo et al. [15] bring attention to the high dietary risk in older stroke patients, showing a correlation between poorer functional outcomes and increased inflammation. This aligns with the growing evidence that nutritional risk is not just a matter of dietary intake but also a significant factor influencing the rehabilitation process, functional recovery, and overall prognosis.

Additionally, the role of micronutrients, such as vitamin D and B-complex vitamins, in cognitive function and neurological recovery is becoming increasingly evident, as noted by Chailurkit et al. [18] and Smith et al. [19]. These micronutrients play a critical role in neural function, and their deficiency or insufficiency can significantly hamper recovery processes. Therefore, regular assessments of these micronutrients should be an integral part of nutritional management in stroke survivors.

Despite these promising results, the variation in study designs, populations, and interventions warrants cautious interpretation. The heterogeneity of these studies reflects the complexity of stroke rehabilitation, underscoring the need for further research with larger sample sizes and consistent measures. Such research is essential to establish more definitive connections between specific nutritional interventions and functional outcomes.

This review not only confirms the profound connection between nutrition and stroke rehabilitation but also advocates for the integration of tailored nutritional interventions in rehabilitation protocols. The evidence suggests that a comprehensive approach to nutritional care, including early intervention, individualized support, and regular assessment of both macro- and micronutrient statuses, can significantly enhance functional recovery in stroke patients. Further research is imperative to refine nutritional care guidelines and optimize rehabilitation strategies in stroke care [17-19].

## Conclusions

This systematic review highlights the significant impact of nutritional interventions on functional recovery in stroke rehabilitation. The evidence from the studies reviewed underscores the importance of early dietary assessment and intervention, particularly for older stroke patients and those at high nutritional risk. Sarcopenia, often overlooked, emerges as a critical factor influencing rehabilitation outcomes. In the critical early stages of stroke recovery, early identification and management of nutritional needs, coupled with ensuring sufficient energy and protein intake, are crucial for bolstering functional independence. This involves improving a patient's ability to perform daily activities autonomously, thereby significantly raising the chances of a successful return to their home environment.

Despite the promising findings, the heterogeneity of study designs and methodologies calls for further research with standardized approaches. This is essential for developing clear guidelines on integrating nutritional interventions into stroke rehabilitation protocols. As healthcare professionals strive for improved care, incorporating personalized nutritional strategies alongside traditional rehabilitation methods is crucial. This holistic approach will ensure stroke survivors receive the most effective and comprehensive rehabilitation, enhancing their overall quality of life.
